# 3-Cyclo­hexyl­sulfonyl-5-iodo-2,7-dimethyl-1-benzofuran

**DOI:** 10.1107/S1600536811027395

**Published:** 2011-07-16

**Authors:** Pil Ja Seo, Hong Dae Choi, Byeng Wha Son, Uk Lee

**Affiliations:** a Department of Chemistry, Dongeui University, San 24 Kaya-dong Busanjin-gu, Busan 614-714, Republic of Korea; b Department of Chemistry, Pukyong National University, 599-1 Daeyeon 3-dong, Nam-gu, Busan 608-737, Republic of Korea

## Abstract

In the title compound, C_16_H_19_IO_3_S, the cyclo­hexyl ring adopts a chair conformation. In the crystal, pairs of inter­molecular I⋯O contacts [3.269 (2) Å] link the mol­ecules into inversion dimers. These dimers are further stabilized by a slipped π–π inter­action between the benzene and furan rings of adjacent mol­ecules [centroid–centroid distance = 3.701 (3) Å, inter­planar distance = 3.372 (3) Å and slippage = 1.525 (3) Å].

## Related literature

For the pharmacological activity of benzofuran compounds, see: Aslam *et al.* (2009 ▶); Galal *et al.* (2009 ▶); Khan *et al.* (2005 ▶). For natural products with benzofuran rings, see: Akgul & Anil (2003 ▶); Soekamto *et al.* (2003 ▶). For structural studies of related 3-cyclo­hexyl­sulfonyl-5-halo-2-methyl-1-benzofuran derivatives, see: Choi *et al.* (2011**a* ▶,*b* ▶,c* ▶). For a review on halogen bonding, see: Politzer *et al.* (2007 ▶).
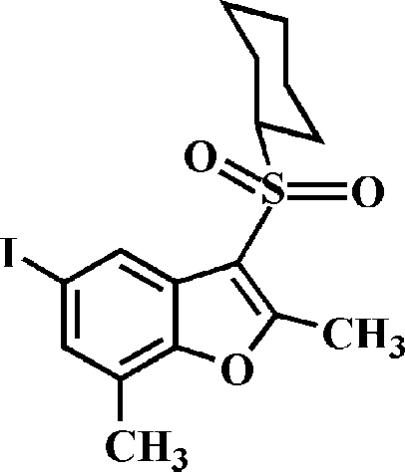


## Experimental

### 

#### Crystal data

C_16_H_19_IO_3_S
*M* *_r_* = 418.27
Triclinic, 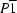

*a* = 6.8643 (2) Å
*b* = 8.4981 (2) Å
*c* = 14.2323 (3) Å
α = 102.512 (1)°
β = 99.846 (1)°
γ = 92.092 (1)°
*V* = 796.23 (3) Å^3^
*Z* = 2
Mo *K*α radiation
μ = 2.15 mm^−1^
*T* = 173 K
0.24 × 0.16 × 0.14 mm

#### Data collection

Bruker SMART APEXII CCD diffractometer
Absorption correction: multi-scan (*SADABS*; Bruker, 2009 ▶) *T*_min_ = 0.629, *T*_max_ = 0.759
15021 measured reflections
3997 independent reflections
3753 reflections with *I* > 2σ(*I*)
*R*_int_ = 0.035

#### Refinement

*R*[*F* ^2^ > 2σ(*F* ^2^)] = 0.023
*wR*(*F* ^2^) = 0.058
*S* = 1.06
3997 reflections
192 parameters
H-atom parameters constrained
Δρ_max_ = 0.68 e Å^−3^
Δρ_min_ = −0.63 e Å^−3^

### 

Data collection: *APEX2* (Bruker, 2009 ▶); cell refinement: *SAINT* (Bruker, 2009 ▶); data reduction: *SAINT*; program(s) used to solve structure: *SHELXS97* (Sheldrick, 2008 ▶); program(s) used to refine structure: *SHELXL97* (Sheldrick, 2008 ▶); molecular graphics: *ORTEP-3* (Farrugia, 1997 ▶) and *DIAMOND* (Brandenburg, 1998 ▶); software used to prepare material for publication: *SHELXL97*.

## Supplementary Material

Crystal structure: contains datablock(s) global, I. DOI: 10.1107/S1600536811027395/zl2384sup1.cif

Structure factors: contains datablock(s) I. DOI: 10.1107/S1600536811027395/zl2384Isup2.hkl

Supplementary material file. DOI: 10.1107/S1600536811027395/zl2384Isup3.cml

Additional supplementary materials:  crystallographic information; 3D view; checkCIF report

## Figures and Tables

**Fig. 1.**
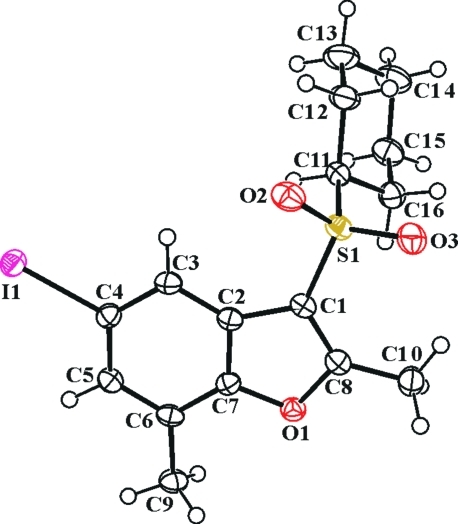
The molecular structure of the title compound with the atom numbering scheme. Displacement ellipsoids are drawn at the 50% probability level. H atoms are presented as a small spheres of arbitrary radius.

**Fig. 2.**
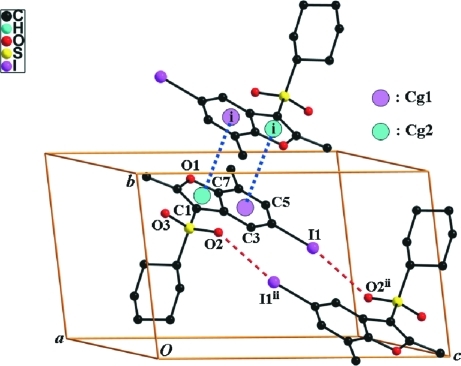
A view of the I···O and π···π interactions (dotted lines) in the crystal structure of the title compound. [Symmetry codes: (i) - x + 1, - y + 2, - z + 1; (ii) - x, - y + 1, - z + 1.]

## supplementary crystallographic information

### Comment

Recently, many compounds containing a benzofuran moiety have drawn much attention owing to their valuable pharmacological properties such as antibacterial and antifungal, antitumor and antiviral, and antimicrobial activities (Aslam *et al.*, 2009, Galal *et al.*, 2009, Khan *et al.*, 2005). These benzofuran derivatives occur in a wide range of natural products (Akgul & Anil, 2003; Soekamto *et al.*, 2003). As a part of our ongoing study of the substituent effect on the solid state structures of 3-cyclohexylsulfonyl-5-halo-2-methyl-1-benzofuran analogues (Choi *et al.*, 2011**a*,*b*,c*), we report herein the crystal structure of the title compound.

In the title molecule (Fig. 1), the benzofuran unit is essentially planar, with a mean deviation of 0.007 (2) Å from the least-squares plane defined by the nine constituent atoms. The cyclohexyl ring is in the chair form. The molecular packing (Fig. 2) is stabilized by I···O halogen-bondings between the iodine and the O atom of the sulfonyl group [I1···O2^ii^ = 3.269 (2) Å; C4—11···O2^ii^ = 168.48 (6)°] (Politzer  *et al.*, 2007). The crystal packing (Fig. 2) is further stabilized by a weak slipped π···π interaction between the benzene and furan rings of adjacent molecules, with a Cg1···Cg2^i^ distance of 3.701 (3) Å and an interplanar distance of 3.372 (3) Å resulting in a slippage of 1.525 (3) Å (Cg1 and Cg2 are the centroids of the C2–C7 benzene ring and the C1/C2/C7/O1/C8 furan ring, respectively, see Fig. 2 for symmetry codes).

### Experimental

77% 3-chloroperoxybenzoic acid (426 mg, 1.9 mmol) was added in small portions to a stirred solution of 3-cyclohexylsulfanyl-5-iodo-2,7-dimethyl-1-benzofuran (347 mg, 0.9 mmol) in dichloromethane (35 mL) at 273 K. After being stirred at room temperature for 8h, the mixture was washed with saturated sodium bicarbonate solution and the organic layer was separated, dried over magnesium sulfate, filtered and concentrated at reduced pressure. The residue was purified by column chromatography (hexane–ethyl acetate, 4:1 v/v) to afford the title compound as a colorless solid [yield 71%, m.p. 441–442 K; *R*_f_ = 0.63 (hexane–ethyl acetate, 4:1 v/v)]. Single crystals suitable for X-ray diffraction were prepared by slow evaporation of a solution of the title compound in acetone at room temperature.

### Refinement

All H atoms were positioned geometrically and refined using a riding model, with C—H = 0.95 Å for aryl, 1.00 Å for methine, 0.99 Å for methylene and 0.98 Å for methyl H atoms, respectively. *U*_iso_(H)  = 1.2*U*_eq_(C) for aryl,  methine and methylene, and 1.5*U*_eq_(C) for methyl H atoms.

### Crystal data

**Table:**

C_16_H_19_IO_3_S	*Z* = 2
*M**_r_* = 418.27	*F*(000) = 416
Triclinic, *P*1	*D*_x_ = 1.745 Mg m^−^^3^
Hall symbol: -P 1	Mo *K*α radiation, λ = 0.71073 Å
*a* = 6.8643 (2) Å	Cell parameters from 8926 reflections
*b* = 8.4981 (2) Å	θ = 2.5–28.4°
*c* = 14.2323 (3) Å	µ = 2.15 mm^−^^1^
α = 102.512 (1)°	*T* = 173 K
β = 99.846 (1)°	Block, colourless
γ = 92.092 (1)°	0.24 × 0.16 × 0.14 mm
*V* = 796.23 (3) Å^3^	

### Data collection

**Table:**

Bruker SMART APEXII CCD diffractometer	3997 independent reflections
Radiation source: rotating anode	3753 reflections with *I* > 2σ(*I*)
graphite multilayer	*R*_int_ = 0.035
Detector resolution: 10.0 pixels mm^-1^	θ_max_ = 28.4°, θ_min_ = 1.5°
φ and ω scans	*h* = −7→9
Absorption correction: multi-scan (*SADABS*; Bruker, 2009)	*k* = −11→11
*T*_min_ = 0.629, *T*_max_ = 0.759	*l* = −19→18
15021 measured reflections	

### Refinement

**Table:**

Refinement on *F*^2^	Primary atom site location: structure-invariant direct methods
Least-squares matrix: full	Secondary atom site location: difference Fourier map
*R*[*F*^2^ > 2σ(*F*^2^)] = 0.023	Hydrogen site location: difference Fourier map
*wR*(*F*^2^) = 0.058	H-atom parameters constrained
*S* = 1.06	*w* = 1/[σ^2^(*F*_o_^2^) + (0.0291*P*)^2^ + 0.2895*P*] where *P* = (*F*_o_^2^ + 2*F*_c_^2^)/3
3997 reflections	(Δ/σ)_max_ = 0.003
192 parameters	Δρ_max_ = 0.68 e Å^−^^3^
0 restraints	Δρ_min_ = −0.63 e Å^−^^3^

### Special details

**Table:**

Geometry. All esds (except the esd in the dihedral angle between two l.s. planes) are estimated using the full covariance matrix. The cell esds are taken into account individually in the estimation of esds in distances, angles and torsion angles; correlations between esds in cell parameters are only used when they are defined by crystal symmetry. An approximate (isotropic) treatment of cell esds is used for estimating esds involving l.s. planes.
Refinement. Refinement of F^2^ against ALL reflections. The weighted R-factor wR and goodness of fit S are based on F^2^, conventional R-factors R are based on F, with F set to zero for negative F^2^. The threshold expression of F^2^ > 2sigma(F^2^) is used only for calculating R-factors(gt) etc. and is not relevant to the choice of reflections for refinement. R-factors based on F^2^ are statistically about twice as large as those based on F, and R- factors based on ALL data will be even larger.

### Fractional atomic coordinates and isotropic or equivalent isotropic displacement parameters (Å^2^)

**Table:**

	*x*	*y*	*z*	*U*_iso_*/*U*_eq_	
I1	0.227778 (18)	0.565947 (15)	0.643317 (9)	0.02648 (5)	
S1	0.19017 (7)	0.68116 (6)	0.21668 (4)	0.02476 (10)	
O1	0.6820 (2)	0.89122 (16)	0.38987 (10)	0.0237 (3)	
O2	0.0181 (2)	0.67742 (19)	0.26177 (12)	0.0329 (3)	
O3	0.1832 (3)	0.76329 (19)	0.13777 (12)	0.0350 (4)	
C1	0.3897 (3)	0.7652 (2)	0.30960 (14)	0.0224 (4)	
C2	0.4132 (3)	0.7461 (2)	0.40933 (14)	0.0211 (4)	
C3	0.3023 (3)	0.6713 (2)	0.46236 (14)	0.0234 (4)	
H3	0.1761	0.6163	0.4336	0.028*	
C4	0.3852 (3)	0.6808 (2)	0.55982 (14)	0.0229 (4)	
C5	0.5707 (3)	0.7612 (2)	0.60332 (14)	0.0231 (4)	
H5	0.6207	0.7642	0.6702	0.028*	
C6	0.6829 (3)	0.8363 (2)	0.55159 (15)	0.0225 (4)	
C7	0.5962 (3)	0.8262 (2)	0.45489 (14)	0.0211 (4)	
C8	0.5545 (3)	0.8523 (2)	0.30207 (14)	0.0238 (4)	
C9	0.8856 (3)	0.9191 (3)	0.59487 (16)	0.0278 (4)	
H9A	0.9151	0.9214	0.6650	0.042*	
H9B	0.8892	1.0300	0.5855	0.042*	
H9C	0.9846	0.8600	0.5623	0.042*	
C10	0.6225 (3)	0.9118 (3)	0.22258 (16)	0.0311 (4)	
H10A	0.6478	1.0297	0.2421	0.047*	
H10B	0.5201	0.8827	0.1633	0.047*	
H10C	0.7449	0.8628	0.2094	0.047*	
C11	0.2463 (3)	0.4764 (2)	0.17566 (14)	0.0252 (4)	
H11	0.2952	0.4337	0.2349	0.030*	
C12	0.0574 (3)	0.3736 (3)	0.11981 (17)	0.0342 (5)	
H12A	−0.0438	0.3806	0.1622	0.041*	
H12B	0.0030	0.4145	0.0614	0.041*	
C13	0.1052 (4)	0.1980 (3)	0.0884 (2)	0.0461 (6)	
H13A	0.1485	0.1550	0.1473	0.055*	
H13B	−0.0160	0.1324	0.0500	0.055*	
C14	0.2662 (4)	0.1825 (3)	0.0274 (2)	0.0446 (6)	
H14A	0.2180	0.2151	−0.0346	0.054*	
H14B	0.2979	0.0681	0.0112	0.054*	
C15	0.4538 (4)	0.2879 (3)	0.08190 (18)	0.0365 (5)	
H15A	0.5530	0.2811	0.0385	0.044*	
H15B	0.5111	0.2470	0.1399	0.044*	
C16	0.4094 (3)	0.4643 (3)	0.11478 (16)	0.0303 (4)	
H16A	0.5310	0.5283	0.1541	0.036*	
H16B	0.3672	0.5096	0.0567	0.036*	

### Atomic displacement parameters (Å^2^)

**Table:**

	*U*^11^	*U*^22^	*U*^33^	*U*^12^	*U*^13^	*U*^23^
I1	0.02413 (8)	0.02921 (8)	0.02755 (8)	0.00073 (5)	0.00534 (5)	0.00927 (5)
S1	0.0237 (2)	0.0259 (2)	0.0223 (2)	0.00194 (18)	−0.00089 (18)	0.00417 (18)
O1	0.0210 (7)	0.0248 (7)	0.0242 (7)	−0.0001 (5)	0.0038 (5)	0.0039 (5)
O2	0.0221 (7)	0.0389 (9)	0.0338 (8)	0.0025 (6)	0.0019 (6)	0.0022 (6)
O3	0.0392 (9)	0.0337 (8)	0.0312 (8)	0.0025 (7)	−0.0044 (7)	0.0138 (6)
C1	0.0218 (9)	0.0223 (9)	0.0211 (9)	0.0025 (7)	0.0017 (7)	0.0024 (7)
C2	0.0201 (9)	0.0189 (8)	0.0227 (9)	0.0030 (7)	0.0030 (7)	0.0020 (7)
C3	0.0197 (9)	0.0227 (9)	0.0256 (9)	−0.0006 (7)	0.0022 (7)	0.0024 (7)
C4	0.0226 (9)	0.0216 (9)	0.0254 (9)	0.0019 (7)	0.0064 (7)	0.0054 (7)
C5	0.0224 (9)	0.0237 (9)	0.0224 (9)	0.0041 (7)	0.0017 (7)	0.0046 (7)
C6	0.0204 (9)	0.0183 (8)	0.0257 (9)	0.0030 (7)	0.0009 (7)	0.0010 (7)
C7	0.0206 (9)	0.0189 (8)	0.0239 (9)	0.0021 (7)	0.0056 (7)	0.0039 (7)
C8	0.0249 (9)	0.0223 (9)	0.0230 (9)	0.0046 (7)	0.0025 (7)	0.0036 (7)
C9	0.0202 (9)	0.0300 (10)	0.0302 (10)	−0.0015 (8)	−0.0014 (8)	0.0054 (8)
C10	0.0329 (11)	0.0346 (11)	0.0277 (10)	0.0014 (9)	0.0071 (9)	0.0100 (8)
C11	0.0289 (10)	0.0245 (9)	0.0203 (9)	−0.0009 (8)	0.0019 (8)	0.0036 (7)
C12	0.0302 (11)	0.0368 (12)	0.0294 (11)	−0.0073 (9)	0.0045 (9)	−0.0034 (9)
C13	0.0499 (16)	0.0352 (13)	0.0436 (14)	−0.0134 (11)	0.0089 (12)	−0.0092 (10)
C14	0.0479 (15)	0.0363 (13)	0.0398 (13)	−0.0014 (11)	0.0066 (11)	−0.0106 (10)
C15	0.0369 (13)	0.0331 (12)	0.0353 (12)	0.0053 (9)	0.0057 (10)	−0.0009 (9)
C16	0.0304 (11)	0.0312 (11)	0.0286 (10)	0.0011 (8)	0.0068 (9)	0.0047 (8)

### Geometric parameters (Å, °)

**Table:**

I1—C4	2.097 (2)	C9—H9C	0.9800
I1—O2^i^	3.2688 (17)	C10—H10A	0.9800
S1—O2	1.4401 (17)	C10—H10B	0.9800
S1—O3	1.4403 (16)	C10—H10C	0.9800
S1—C1	1.742 (2)	C11—C16	1.522 (3)
S1—C11	1.790 (2)	C11—C12	1.530 (3)
O1—C8	1.365 (2)	C11—H11	1.0000
O1—C7	1.376 (2)	C12—C13	1.527 (3)
C1—C8	1.361 (3)	C12—H12A	0.9900
C1—C2	1.446 (3)	C12—H12B	0.9900
C2—C3	1.386 (3)	C13—C14	1.510 (4)
C2—C7	1.389 (3)	C13—H13A	0.9900
C3—C4	1.390 (3)	C13—H13B	0.9900
C3—H3	0.9500	C14—C15	1.528 (3)
C4—C5	1.398 (3)	C14—H14A	0.9900
C5—C6	1.380 (3)	C14—H14B	0.9900
C5—H5	0.9500	C15—C16	1.529 (3)
C6—C7	1.386 (3)	C15—H15A	0.9900
C6—C9	1.503 (3)	C15—H15B	0.9900
C8—C10	1.472 (3)	C16—H16A	0.9900
C9—H9A	0.9800	C16—H16B	0.9900
C9—H9B	0.9800		
			
C4—I1—O2^i^	168.48 (6)	H10A—C10—H10B	109.5
O2—S1—O3	118.27 (10)	C8—C10—H10C	109.5
O2—S1—C1	107.00 (9)	H10A—C10—H10C	109.5
O3—S1—C1	109.53 (10)	H10B—C10—H10C	109.5
O2—S1—C11	107.41 (10)	C16—C11—C12	111.79 (17)
O3—S1—C11	109.40 (10)	C16—C11—S1	111.94 (14)
C1—S1—C11	104.32 (9)	C12—C11—S1	109.81 (15)
C8—O1—C7	107.13 (15)	C16—C11—H11	107.7
C8—C1—C2	107.46 (17)	C12—C11—H11	107.7
C8—C1—S1	127.49 (16)	S1—C11—H11	107.7
C2—C1—S1	124.98 (15)	C13—C12—C11	109.4 (2)
C3—C2—C7	119.59 (18)	C13—C12—H12A	109.8
C3—C2—C1	135.79 (18)	C11—C12—H12A	109.8
C7—C2—C1	104.61 (17)	C13—C12—H12B	109.8
C2—C3—C4	116.60 (18)	C11—C12—H12B	109.8
C2—C3—H3	121.7	H12A—C12—H12B	108.2
C4—C3—H3	121.7	C14—C13—C12	111.6 (2)
C3—C4—C5	122.42 (19)	C14—C13—H13A	109.3
C3—C4—I1	118.52 (14)	C12—C13—H13A	109.3
C5—C4—I1	119.05 (14)	C14—C13—H13B	109.3
C6—C5—C4	121.71 (18)	C12—C13—H13B	109.3
C6—C5—H5	119.1	H13A—C13—H13B	108.0
C4—C5—H5	119.1	C13—C14—C15	111.3 (2)
C5—C6—C7	114.71 (17)	C13—C14—H14A	109.4
C5—C6—C9	123.24 (19)	C15—C14—H14A	109.4
C7—C6—C9	122.03 (19)	C13—C14—H14B	109.4
O1—C7—C6	124.57 (17)	C15—C14—H14B	109.4
O1—C7—C2	110.47 (16)	H14A—C14—H14B	108.0
C6—C7—C2	124.95 (19)	C14—C15—C16	111.3 (2)
C1—C8—O1	110.33 (17)	C14—C15—H15A	109.4
C1—C8—C10	134.78 (19)	C16—C15—H15A	109.4
O1—C8—C10	114.89 (18)	C14—C15—H15B	109.4
C6—C9—H9A	109.5	C16—C15—H15B	109.4
C6—C9—H9B	109.5	H15A—C15—H15B	108.0
H9A—C9—H9B	109.5	C11—C16—C15	110.27 (19)
C6—C9—H9C	109.5	C11—C16—H16A	109.6
H9A—C9—H9C	109.5	C15—C16—H16A	109.6
H9B—C9—H9C	109.5	C11—C16—H16B	109.6
C8—C10—H10A	109.5	C15—C16—H16B	109.6
C8—C10—H10B	109.5	H16A—C16—H16B	108.1
			
O2—S1—C1—C8	−150.57 (18)	C9—C6—C7—C2	177.36 (18)
O3—S1—C1—C8	−21.2 (2)	C3—C2—C7—O1	179.81 (16)
C11—S1—C1—C8	95.78 (19)	C1—C2—C7—O1	0.2 (2)
O2—S1—C1—C2	32.94 (19)	C3—C2—C7—C6	1.2 (3)
O3—S1—C1—C2	162.28 (16)	C1—C2—C7—C6	−178.46 (18)
C11—S1—C1—C2	−80.70 (18)	C2—C1—C8—O1	−0.2 (2)
C8—C1—C2—C3	−179.5 (2)	S1—C1—C8—O1	−177.21 (14)
S1—C1—C2—C3	−2.4 (3)	C2—C1—C8—C10	179.9 (2)
C8—C1—C2—C7	0.0 (2)	S1—C1—C8—C10	2.9 (4)
S1—C1—C2—C7	177.11 (14)	C7—O1—C8—C1	0.3 (2)
C7—C2—C3—C4	−0.5 (3)	C7—O1—C8—C10	−179.77 (16)
C1—C2—C3—C4	179.0 (2)	O2—S1—C11—C16	172.45 (14)
C2—C3—C4—C5	0.0 (3)	O3—S1—C11—C16	42.91 (17)
C2—C3—C4—I1	−178.70 (13)	C1—S1—C11—C16	−74.20 (16)
O2^i^—I1—C4—C3	54.4 (4)	O2—S1—C11—C12	47.67 (17)
O2^i^—I1—C4—C5	−124.3 (3)	O3—S1—C11—C12	−81.87 (17)
C3—C4—C5—C6	0.0 (3)	C1—S1—C11—C12	161.02 (15)
I1—C4—C5—C6	178.69 (14)	C16—C11—C12—C13	57.2 (3)
C4—C5—C6—C7	0.5 (3)	S1—C11—C12—C13	−177.91 (17)
C4—C5—C6—C9	−177.95 (18)	C11—C12—C13—C14	−56.7 (3)
C8—O1—C7—C6	178.33 (18)	C12—C13—C14—C15	56.3 (3)
C8—O1—C7—C2	−0.3 (2)	C13—C14—C15—C16	−55.2 (3)
C5—C6—C7—O1	−179.56 (17)	C12—C11—C16—C15	−56.8 (2)
C9—C6—C7—O1	−1.1 (3)	S1—C11—C16—C15	179.57 (15)
C5—C6—C7—C2	−1.1 (3)	C14—C15—C16—C11	55.0 (3)

Symmetry codes: (i) −*x*, −*y*+1, −*z*+1.

## References

[bb1] Akgul, Y. Y. & Anil, H. (2003). *Phytochemistry*, **63**, 939–943. 10.1016/s0031-9422(03)00357-1 12895543

[bb2] Aslam, S. N., Stevenson, P. C., Kokubun, T. & Hall, D. R. (2009). *Microbiol. Res.* **164**, 191–195. 10.1016/j.micres.2006.11.012 17418552

[bb3] Brandenburg, K. (1998). *DIAMOND* Crystal Impact GbR, Bonn, Germany.

[bb4] Bruker (2009). *APEX2*, *SADABS* and *SAINT* Bruker AXS Inc., Madison, Wisconsin, USA.

[bb5] Choi, H. D., Seo, P. J., Son, B. W. & Lee, U. (2011*a*). *Acta Cryst.* E**67**, o542. 10.1107/S1600536811003515 PMC3052108 21522310

[bb6] Choi, H. D., Seo, P. J., Son, B. W. & Lee, U. (2011*b*). *Acta Cryst.* E**67**, o828. 10.1107/S1600536811008270 PMC3099810 21754112

[bb7] Choi, H. D., Seo, P. J., Son, B. W. & Lee, U. (2011*c*). *Acta Cryst.* E**67**, o847. 10.1107/S1600536811008580 PMC3099950 21754129

[bb8] Farrugia, L. J. (1997). *J. Appl. Cryst.* **30**, 565.

[bb9] Galal, S. A., Abd El-All, A. S., Abdallah, M. M. & El-Diwani, H. I. (2009). *Bioorg. Med. Chem. Lett* **19**, 2420–2428. 10.1016/j.bmcl.2009.03.069 19345581

[bb10] Khan, M. W., Alam, M. J., Rashid, M. A. & Chowdhury, R. (2005). *Bioorg. Med. Chem* **13**, 4796–4805. 10.1016/j.bmc.2005.05.009 15964760

[bb11] Politzer, P., Lane, P., Concha, M. C., Ma, Y. & Murray, J. S. (2007). *J. Mol. Model* **13**, 305–311. 10.1007/s00894-006-0154-7 17013631

[bb12] Sheldrick, G. M. (2008). *Acta Cryst.* A**64**, 112–122. 10.1107/S0108767307043930 18156677

[bb13] Soekamto, N. H., Achmad, S. A., Ghisalberti, E. L., Hakim, E. H. & Syah, Y. M. (2003). *Phytochemistry*, **64**, 831–834. 10.1016/j.phytochem.2003.08.009 14559276

